# Cooled Radiofrequency Treatment for Radicular Pain Related to Lumbar Disc Herniation

**DOI:** 10.7759/cureus.46255

**Published:** 2023-09-30

**Authors:** Juan J Medina-Pérez, Andrés Vega-Rosas, Silvia G Coubert-Pelayo, Lissa S Rosas-Barcelo

**Affiliations:** 1 Pain Management, Hospital Ángeles Mocel, Mexico City, MEX

**Keywords:** radio frequency, discogenic back pain, painful neuropathy, lumbar disc hernia, chronic low back pain (clbp)

## Abstract

Background: Lower back and radicular pain are leading causes of disability and loss of quality of life, especially due to its high prevalence in the general population. Cooled radiofrequency treatment (CRT) has emerged as a novel non-invasive technique for the management of discogenic pain with safe and effective results. The aim of this study was to evaluate the effectiveness of CRT in the treatment of radicular pain secondary to a lumbar hernia in patients with chronic neuropathic pain after receiving conservative treatment that did not achieve adequate pain management.

Methods: A prospective study of patients undergoing CRT for the management of discogenic low back pain was carried out. When attending the first evaluation and corroborating the lumbar hernias by magnetic resonance imaging (MRI), treatment was offered one month of physiotherapy before CRT. To assess the evolution of the patients, measurements were taken before and after the intervention with the visual analog scale (VAS) and the Oswestry low back pain disability scale (OLBPDS) scores.

Results: A total of 74 patients (mean age: 48.42 ± 14.32 years, 66.11% female) were included, who were undergoing a total of 134 herniated intervertebral lumbar discs. When comparing the initial perception of low back pain and after finding a non-significant partial improvement with one month of physiotherapy treatment, it was observed that the patients who were offered CRT showed an average improvement in discogenic pain of 79.92% (p = <0.0001, 95% CI: -7.010 to -6.379) in 98.64% of cases. This was accompanied by an increase in their functionality of daily living activities, as measured by OLBPDS. No patients presented significant adverse events, and in the only case where the desired pain management was not obtained, the patient's discomfort did not worsen.

Conclusions: Intradiscal biacuplasty by CRT is a considerable treatment for lumbar radiculopathy. Postoperative results demonstrated its effectiveness and safety in the management of radicular pain without the presence of significant adverse effects.

## Introduction

Low back pain is a leading cause of disability and productivity loss, associated with high costs and some of the most frequent reasons for visiting a general practitioner [1,2], so adequate evidence-based management turns out to be a priority [3]. A systematic review of the global prevalence of low back pain reported a prevalence estimated to be 11.9 ± 2.0%, and the one-month prevalence was estimated to be 23.2 ± 2.9%, predominantly affecting female individuals and those aged 40-80 years [4]. They are generally characterized by different types of pain, such as nociceptive, neuropathic (radicular), and nociplastic or non-specific, which frequently overlap after a stressor agent toward any of the elements that make up the lumbar spine [5], being the post-traumatic herniated discs one of the most important etiologies.

Lumbar post-traumatic herniated disc is one of the main causes of discogenic back pain as it causes compression of the nerve root. As there is a compromise of the nervous system, it generates more complex clinical presentations that are difficult to explain by simple mechanical compromise. Within the processes that occur in pain due to a herniated disc are the inflammatory cascades [6] and the neuromodulation systems, which are increasingly understood. Specifically for low back pain, the European clinical guidelines recommended entirely non‐pharmacological treatments, based on stronger evidence; while psychological therapies, multidisciplinary treatment, and referral for surgery were recommended for specific subgroups [3]. The most common surgical treatment for lumbar radiculopathy or sciatica due to lumbar disc herniation is the lumbar discectomy with roughly 180,000-200,000 cases performed annually in the United States. Discectomy has shown better functional outcomes and pain scores in the short-term, but by two- to four-year follow-up, statistical significance is lost, in addition to adverse effects such as cauda equina syndrome, postlaminectomy syndrome, or progressive neurologic deficit [7,8]. A recent meta-analysis of a total of 19 articles that involved 2272 participants concluded low-quality evidence, which suggested that surgical treatment is more effective than nonoperative treatment in improving physical functions [9].

With technological progress, less invasive therapeutic strategies have been sought that offer better clinical results. Intradiscal electrothermal therapy has gained great relevance in the last decade since it is a procedure that may eliminate or delay the need for surgical intervention for an extended period in properly selected patients, whereas only a few adverse effects have been reported [10]. Intradiscal biacuplasty by cooled radiofrequency treatment (CRT) is a non-opioid procedure that works by transmitting thermal energy that causes the ablation of the target tissue (such as the hernia that generates compression of the nerve root), ensuring that healthy tissue is not affected through the precision of a water-cooling system that prevents the device from heating outside the tip of the electrode, reducing the absorption of energy in the other anatomical structures. The aim of this study was to evaluate the effectiveness of CRT in the treatment of radicular pain secondary to a lumbar hernia in patients with chronic neuropathic pain after receiving conservative treatment that did not achieve adequate pain management.

## Materials and methods

Study design

A crossover clinical trial was carried out, where the patients with chronic radicular pain secondary to lumbar disc herniation, who had received previous conservative treatment and a month of physiotherapy without achieving adequate pain control, underwent CRT in the discs that presented hernias. It evaluated the VAS and the OLBPDS pre- and postintervention. After the initial clinical evaluation by a physician specializing in interventional pain medicine, physical therapy treatment with a certified therapist was offered for one month. Patients who did not show significant clinical improvement were offered CRT intervention. Subsequently, the patients were summoned to monitor the evolution of the pain and functionality by applying the aforementioned scales again. In accordance with the Declaration of Helsinki and Good Clinical Practices, each patient was informed about the procedure after being selected followed by a specialized medical evaluation by a certified algologist, who explained the risks and benefits, which were accepted by the patients through a signed and dated informed consent.

Patients

Adult subjects with a history of discogenic lumbar back pain for at least three months were included. The patients to be selected had to present back pain of intensity ≥ 7/10 points on the visual analog scale (VAS) and moderate or severe score on the Oswestry low back pain disability scale (OLBPDS), express their desire to receive the intervention, have previously subjected to conventional treatments such as analgesics or physical therapy without success, and have documented the presence of disc herniations by means of magnetic resonance imaging (MRI).

Initial physiotherapy approach

Treatment was started with objectives to control pain and promote muscle relaxation, relieve pressure on pain-sensitive or neurological structures, strengthen muscle, reduce paresthesias in the lower limb, and improve postural hygiene. We worked with physical agents such as thermotherapy, electrotherapy, and manual therapy to control pain, with patients who had a posterior or posterolateral protrusion. The therapist worked with passive extension exercise, correction of lateral deviation, and stretching of the soft tissues anterior to the lumbar spine (to increase trunk extension). If the protrusion was anterior, it was worked with passive flexion exercises and stretching of the lumbar erector spinae muscles and the posterior soft tissues of the spine (increased trunk flexion). Reeducation and strengthening of the lumbar extensors as trunk stabilizers and increased muscular resistance to control the spine, as well as stretching of the shortened muscles of the lower extremities that affect posture, were carried out. All patients were re-educated in postural hygiene for their activities of daily living, and active break techniques were implemented in patients who had work activities.

Intradiscal biacuplasty

**Figure 1:**
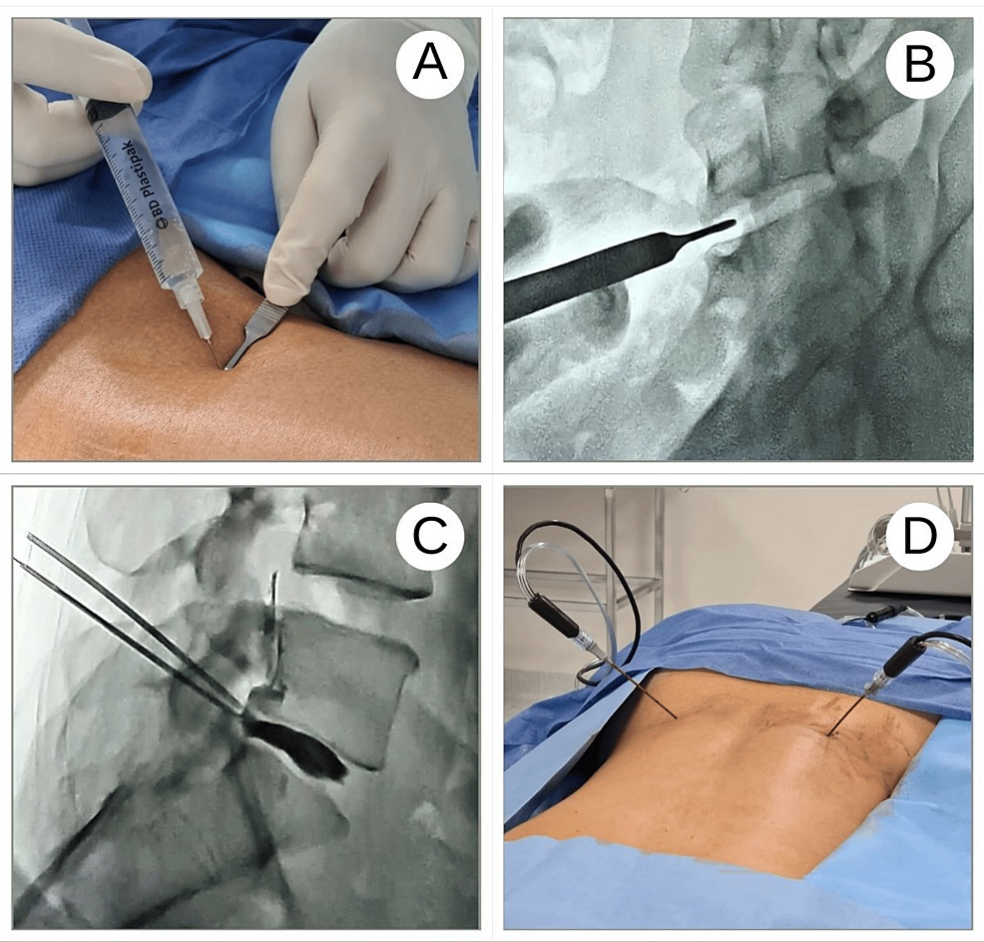
Surgical approach for the administration of intradiscal radiofrequency (A) Infiltration with local anesthesia is performed at the site to be operated on. (B) Using fluoroscopy in a C-arm, the intervertebral space where the hernia is located is corroborated. (C) Discography is performed by injecting a contrast medium to document the presence of pain and the morphological characteristics of the hernia. (D) The intradiscal stimulator is inserted, sensory safety tests are performed with the patient awake, and decompressive radiofrequency is performed.

A COOLIEF® (Alpharetta, Georgia) radiofrequency electrode is used for the procedure, which is connected to the generator and peristaltic pump unit for energy supply and internal cooling. The radiofrequency ablation process is carried out with the tip of the probe at 60°C, while the temperature of the target tissues reaches 80°C, ensuring no damage to other surrounding structures through the cooling system of the probe. A radiopaque marker is located at the proximal end of the active tip; this marker defines the location of the lesion under fluoroscopy, confirming the position and improving visualization. After signing the informed consent, in the operating room with C-arm, the patients were positioned in the prone position with sterile technique through the asepsis and antisepsis protocol to initiate intradiscal biacuplasty (Figure 1).

Skin and deep tissues are infiltrated with 1% lidocaine at the level of the affected disc. The interdiscal spaces of the area of ​​interest are identified in oblique projection at 30º by fluoroscopy, as well as facet joints. With tunnel vision, a supra-pedicular approach was performed through the herniated disc, placing a working channel in the posterior third of the intervertebral disc. Diagnostic evoked discography is performed, which is considered positive if the patient reports pain at the time, corroborating disc herniations. Lumbar decompression is performed, in response to the evoked discography and neurotomy of the gray branches of the corresponding disc with intradiscal bipolar energy bilaterally at 80°C for 15 minutes, after a motor and sensory safety protocol. The event ends by corroborating with the patient's sensitivity and perception of pain, intentionally interrogating incidents or immediate adverse effects. The procedure is performed under local anesthesia and partial sedation, thus keeping the patient conscious for feedback on their somatic perception at all times. The patient remained hospitalized for 24 hours after the intervention for observation and was discharged with oral analgesics and follow-up appointments for interventional pain management and physiotherapy to facilitate timely physical rehabilitation.

Statistical analysis

Continuous demographic data were summarized using descriptive statistics, and the subject was compared with its own control by two-way ANOVA, comparing the initial VAS score, the one after physiotherapy treatment for one month and the subsequent one after undergoing CRT. Categorical demographic data were summarized and expressed as percentages. To determine the homogeneity of the age of the patients, the one-sample t-test and Wilcoxon test were performed. Data comparisons were performed at a 5% level of significance (p ≤ 0.05). In some cases, not every eligible subject provided observations at each follow-up point. GraphPad Prism 9 (GraphPad, La Jolla, California) for macOS software was used for the analysis.

## Results

A total of 106 patients were recruited; however, 74 of them met all inclusion criteria, received physiotherapy treatment for one month without significant improvement, and underwent intradiscal biacuplasty by CRT; the rest were excluded from this study. It should be noted that several patients received CRT at two or three lumbar spinal levels per intervention, resulting in a total of 134 herniated intervertebral discs that were treated. As shown in Table 1, the average age was 49.03 ± 15.15 years, with 25 males (33.78%) and 49 females (66.22%). Additionally, comorbidities were searched, finding that eight subjects had type 2 diabetes mellitus (13.55%), 20 systemic had arterial hypertension (33.89%), and 21 were overweight or obese (35.59%) (Table 1).

**Table 1:** Baseline characteristics of the participants (N = 74)

Demographic parameters	Total	Percentage (%)
Sex		
Female	49	66.22
Male	25	33.78
Mean age (years)	49.03	
Type 2 diabetes mellitus	8	13.55
Arterial hypertension	20	33.89
Overweight or obesity	21	35.59

Table 2 shows the distribution of hernias, indicating that L3-L4 affected 14 (13.33%), L4-L5 affected 49 (46.66%), and L5-S1 affected 42 (40%).

**Table 2:** Anatomical distribution of treated hernias (N = 134)

Vertebral disc	Total	Percentage (%)
L3-L4	18	13.33
L4-L5	63	46.66
L5-S1	53	40

In the evaluation of the VAS, 74 subjects were considered for intervention after reporting a VAS score equal to or greater than 7 points in their initial evaluation by pain medicine. After physical therapy treatment, a vast majority of patients mentioned having a partial improvement in their pain and functionality; however, it was not significant (p = 0.1324, 95% CI: 0.4805 to 0.06380). On the other hand, in the initial evaluation, 54 (72.88%) subjects reported a moderate OLBPDS, and 20 (27.11%) reported a severe one. Regarding the laterality of neuropathic pain, 29 (38.98%) subjects reported that it was unilateral, and 45 (61.02%) reported that it was bilateral. All the patients who underwent surgery presented a post-traumatic etiology of their pain, which had an evolution time between three months and five years, with an average of 11.83 ± 12.1 months between the triggering event and the intradiscal biacuplasty. Regarding previous treatment, 72.88% of subjects were receiving non-opioid analgesics, 10.16% were receiving opioid treatment, and 16.94% received physical therapy on all occasions without obtaining significant pain improvement; none of them had seen a pain specialist.

**Figure 2:**
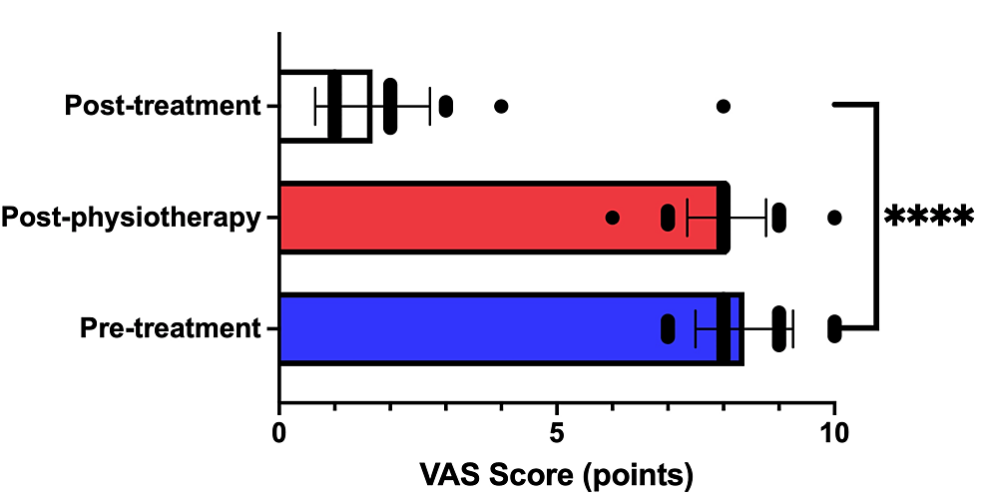
VAS scores before and after the interventions The subjective perception of pain was determined in the first assessment by pain medicine. Subsequently, after a month of initial conservative treatment with physiotherapy, a new evaluation was carried out without finding significant improvement in pain, so they were considered for CRT. After biacuplasty, adequate pain management is verified by finding a statistically significant difference. **** p ≤ 0.0001. CRT: Cooled radiofrequency treatment; VAS: Visual analog scale.

After intradiscal biacuplasty, the patients were followed up between one and six months, obtaining a mean follow-up of 2.42 ± 1.29 months. From the new measurement of the VAS at follow-up, a global improvement of 79.92% of the pain was found (p = <0.0001, 95% CI: 7.010 to -6.379), obtaining adequate pain management in the 98.64% of the cases (Figure 2).

Although there was only one case in which the patient did not report any improvement in radicular pain, during his clinical follow-up, a moderate reduction in lumbar discogenic pain was found; it is important to highlight that there was no worsening of the original painful condition. Relating to the OLBPDS, 98.64% of the patients reported having reached the minimum score, while the patient who did not present improvement in his pain did not increase his functionality significantly either. No patient reported adverse effects other than transient pain in the postoperative period or after their discharge from the hospital. Perhaps, as physiotherapy did not obtain the expected results before CRT, all patients received personalized physical rehabilitation early to accelerate recovery after the intervention under an exercise scheme similar to the previous one, thereby strengthening the clinical outcome.

## Discussion

Back pain is one of the most frequent causes of consultation with primary health providers as it is highly prevalent in the general population and is associated with a worsening of quality of life and mental health throughout life [11,12]. Its early diagnosis and the offer of increasingly less invasive and more effective treatments are a growing need as it predominantly affects adults of working age who are economically active, representing a threat to their financial stability and family well-being, as could be seen in the present study. Radiofrequency has been shown to be an effective non-invasive technique for chronic pain almost immediately after the procedure, with effectiveness and absence of significant complications over time [13-15]. Therefore, it has gained greater confidence for its use, and the availability of this type of technology around the world has improved, especially in Latin America where its growth has been slow but with good results. Within its main clinical applications in the area of ​​pain management, radiofrequency has shown great effectiveness mainly in chronic knee pain due to osteoarthritis [16,17] or geniculate neuropathy [18], which in both cases has been associated with significant improvement in pain and functionality. When the effectiveness of CRT has been compared in preclinical studies against other types of radiofrequency treatments, CRT has shown greater attenuation and prolonged loss of nerve function measured via electromyography [19]. Specifically, clinical reports of intradiscal CRT biacuplasty have been compared with several other pain management techniques, and results have been obtained that indicate significant clinical improvement [20-22], similar to our findings where the algological evaluation by the VAS scale and functional assessment by OLBPDS have shown strong and objective clinical improvement after treatment.

Candan et al. [23] reported that cooled radiofrequency neurotomy for the treatment of chronic lumbar is considered to have a significant clinical impact when patients report pain improvement of at least 50%, finding pain reduction of 60.5% in the first follow-up (four to eight weeks) and 53.6% in the second follow-up (>two to six months), which is consistent with the percentage of pain reduction obtained in the present study. Additionally, due to the lack of adverse effects or severe complications, excluding the expected local pain after the procedure, which in all cases could be controlled with oral analgesics, CRT can be considered a safe procedure. It is important to consider that laminectomy, the most widely used surgical procedure to treat herniated discs, has shown contradictory clinical results regarding the improvement of the VAS score for back and leg pain over time [24] and a reoperation rate of 18% within five years [25]. Regardless of the type of approach, unilateral laminotomy for bilateral decompression, bilateral laminotomy, and split-spinous process laminotomy compared with conventional laminectomy on functional disability, perceived recovery, and leg pain have not shown differences between themselves [26]. In addition, the development of failed back surgery syndrome is common since the failure rates for lumbar fusion range from 30% to 46% and 19% to 25% for microdiscectomy [27]. Therefore, exposing a patient to this risk must be considered after offering all possible conservative and minimally invasive interventions, and that has been refractory. On the other hand, it is interesting to consider how ineffective drug treatment can cause pain to persist and become more complex. Recent evidence suggests that there is a positive correlation between the use of anti-inflammatory drugs and the duration of the deficit regarding sensory function (R = 0.570; p < 0.001) on the expected postsurgical results in a study of cauda equina syndrome associated with herniated discs [28], so maintaining an analgesic treatment of this type of opioid is not an ideal option if a significant improvement is not found.

Considering this, CRT emerges as a therapeutic option with greater effectiveness and a lower risk of severe adverse effects. This technique has been shown to offer statistically superior improvements across the spectrum of patients with chronic sacroiliac joint pain [29], intercostal neuralgia, knee osteoarthritis, discogenic lower back pain [30], and other painful conditions compared to standard medical management. Generally, the improvement of pain by CRT is obtained by ablating the nerves that give rise to the unpleasant sensation. In the present study, it was found that discogenic pain is improved not only by this mechanism but also through the reduction of the volume of the hernia. The nerve root is freed from protrusion, promoting a significant improvement in radicular pain and overall functionality. Despite having a patient whose pain did not improve, the fact that it did not worsen or show any significant adverse effect refers to how safe this minimally invasive technique is, without resulting in a contraindication to later try surgical therapeutic alternatives that could be inefficient and more expensive at the beginning.

The main limitation of this study has been the number of patients and the possibility of comparing the subjects against controls. As it is the first single-center approach in a private hospital in Mexico, the possibility of offering both procedures is complicated; therefore, the selection of patients was made at convenience from the population that voluntarily and autonomously came to this Pain Management Center. Additionally, it is necessary to follow these patients over time and observe how their clinical outcomes have evolved.

## Conclusions

Intradiscal CRT biacuplasty is a safe and effective minimally invasive method for treating chronic neuropathic pain secondary to root compression due to a lumbar hernia and discogenic pain. The subjective perception of pain as measured by the VAS score has decreased significantly after the intervention, improving the quality of life in patients with chronic pain, especially in those who have considered different types of orthopedic spinal interventions or who suffer from pain due to failed back surgery syndrome, after undergoing laminectomies or lumbar instrumentation that failed to provide adequate pain management. On the other hand, the improvement in functionality measured by OLBPDS also demonstrated the superiority of this technique over invasive options. In the case where pain improvement was not achieved, the patient did not show worsening symptoms, not limiting the ability to perform other surgical interventions after conservative and minimally invasive treatment has been refractory, as suggested by the guidelines. More research is needed in the field of algology and pain medicine to offer techniques that are increasingly less invasive, more effective, and have the fewest possible long-term effects.
